# Non-spatial information on the presence of food elevates search intensity in ant workers, leading to faster maze solving in a process parallel to spatial learning

**DOI:** 10.1371/journal.pone.0229709

**Published:** 2020-02-28

**Authors:** Darar Bega, Yehonatan Samocha, Nitzan Yitzhak, Maya Saar, Aziz Subach, Inon Scharf

**Affiliations:** School of Zoology, Faculty of Life Sciences, Tel Aviv University, Tel Aviv, Israel; Wildlife Conservation Society Canada, CANADA

## Abstract

Experience can lead to faster exploitation of food patches through spatial learning or other parallel processes. Past studies have indicated that hungry animals either search more intensively for food or learn better how to detect it. However, fewer studies have examined the contribution of non-spatial information on the presence of food nearby to maze solving, as a parallel process to spatial learning. We exposed *Cataglyphis niger* ant workers to a food reward and then let them search for food in a maze. The information that food existed nearby, even without spatial information, led to faster maze solving compared to a control group that was not exposed to the food prior to the experiment. Faster solving is probably achieved by a higher number of workers entering the maze, following the information that food is present nearby. In a second experiment, we allowed the ants to make successive searches in the maze, followed by removing them after they had returned to the nest and interacted with their naïve nestmates. This procedure led to a maze-solving time in-between that displayed when removing the workers immediately after they had reached the food and preventing their return to the colony, and that of no removal. The workers that interacted upon returning to the nest might have transferred to naïve workers information, unrelated to spatial learning, that food existed nearby, and driven them to commence searching. Spatial learning, or an increase in the correct movements leading to the food reward relative to those leading to dead-ends, was only evident when the same workers were allowed to search again in the same maze. However, both non-spatial information on the presence of food that elevated search intensity and spatial learning led to faster maze solving.

## Introduction

One definition of learning refers to the generated neuronal representations of newly acquired information, which allows an animal to change its responses to specific stimuli or situations [1–2]. Spatial learning is the establishment of memories that enable recognition of locations, dependent on their surroundings, or the procedure by which an animal establishes a mental representation of its environment [3–4]. Many studies have investigated spatial learning and demonstrated the ability of animals to improve their orientation in their habitat with experience [5–10]. This improved orientation contributes to the animal’s fitness. For instance, the spatial learning ability and spatial memory of food-hoarding birds are positively correlated with their survival and reproduction [11–12]. Spatial learning in vertebrates and invertebrates takes place in the hippocampus and mushroom body, respectively [13–16]. Bird populations that heavily rely on spatial memory in their natural habitat usually have larger hippocampi than other populations that rely less on spatial memory [17–18]. Animals use a variety of mechanisms to orient in their environment, depending on their innate abilities, the duration they require to remember specific routes, and the cues available in the environment, such as potential landmarks [2].

Foraging presents a useful context for studying learning, because it takes place consistently throughout an individual’s lifetime, providing opportunities for improvement [19–21]. Learning in this context enables, for example, the faster discovery of prey, faster response to prey, or faster handling of prey [22–24]. However, faster discovery of prey does not mean that only spatial learning is involved in this process. The elevation of different aspects of search intensity can also lead to faster discovery. For example, faster-moving animals detect food more frequently than slow-moving animals, and large groups discover food faster than smaller ones [25–27]. Hunger elevates search intensity as well [28–30], although the pattern that is most reasonable to expect is a hump-shaped pattern, of an initial increase in activity with hunger level, followed by a decrease [31]. The potential effect of hunger level on learning has long been known. Rats, for example, learn faster or err less in a maze when they are hungry and a reward is offered than when they are satiated [32–35]. Unless hunger itself is under study, behavioral studies therefore take care to standardize the hunger level prior to the experiment or account for body condition (e.g., [36–40]). Researchers often standardize prior experience and state (e.g., hunger level) [41–42], but we are not aware of any study demonstrating the effect of non-spatial information on the presence of food, on maze-solving time and consequent food discovery.

Ant colonies have long been the focus of learning studies [43–46]. Ants use various environmental cues and innate abilities, such as a combination of polarized light and step integrator, to estimate their location in respect to the nest; they can also use landmarks and their innate sense of direction in order to choose the shortest path back to the nest, following food discovery [47–50]. Mazes have been traditionally used to test exploration and spatial learning in ants [27, 51–55]. Studies using mazes have demonstrated, for example, that foraging ants can learn a sequence of landmarks that guide them back to the nest in a maze [56–57].

We examined here the possible contribution to later food discovery of non-spatial information on the presence of food, as a parallel process to spatial learning. In two experimental set-ups on ant workers searching in a maze, we allowed information transfer among ant workers on the general presence of food nearby, but without allowing the workers to possess spatial information on its location. We expected elevated search intensity following this exposure to lead to faster discovery of food but expected no change in the spatial information that workers possess. In other words, following exposure to food, either more workers would leave the nest to search in the maze or more workers would move forward in the maze than back in the nest direction. In contrast to the latter two, the ratio between correct movements and wrong ones would not change.

## Materials and methods

### Habitat-of-origin and ant collection and maintenance

We collected 59 colonies of *Cataglyphis niger* from the Tel-Baruch sand dunes in Tel Aviv, Israel (32.132N, 34.788E), a public land. These sand dunes are an enclave of a disturbed natural habitat surrounded by urban areas. *Messor*, *Camponotus*, and other *Cataglyphis* ant species co-occur in the habitat [58–59]. The area comprises stabilized and semi-stabilized dunes, including patches of vegetation [55, 60]. We used this species owing to its individual foraging strategy lacking recruitment and trail pheromones, its occurrence in a variety of habitats, and its medium-sized colonies, which are relatively easy to collect and maintain in the laboratory [61–65]. The fact that *Cataglyphis* spp. are individual foragers indicates that the probability of each worker to discover food is independent of the behavior of other workers. Since *Cataglyphis* ants are ground foragers [66], plants, stones, and debris constitute obstacles, similar to the walls of the mazes we use. Desert ants in general and *Cataglyphis* spp. in particular have excellent navigation skills and use various methods to return to the nest after foraging bouts, such as path integration, dead reckoning, or an acquired panoramic view of the habitat [49, 67–69]. Colonies varied in size from 76 to 721 workers (236 ± 144; mean ± 1 SD) and were evenly distributed across treatments (F_3,55_ = 0.344, P = 0.794). We housed the colonies in Plexiglas nests (20×20×5 cm) in the laboratory (~28°C; 12:12 L:D) and provided them with water in tubes sealed with cotton wool. The experiment started two days after collection (similar to [55]). Following the experiment, we kept them in the laboratory for further research. No permits are required to collect this ant species (unprotected area or species) and the experiment was non-harmful to the ants.

### Basic experimental design

**Fig 1:**
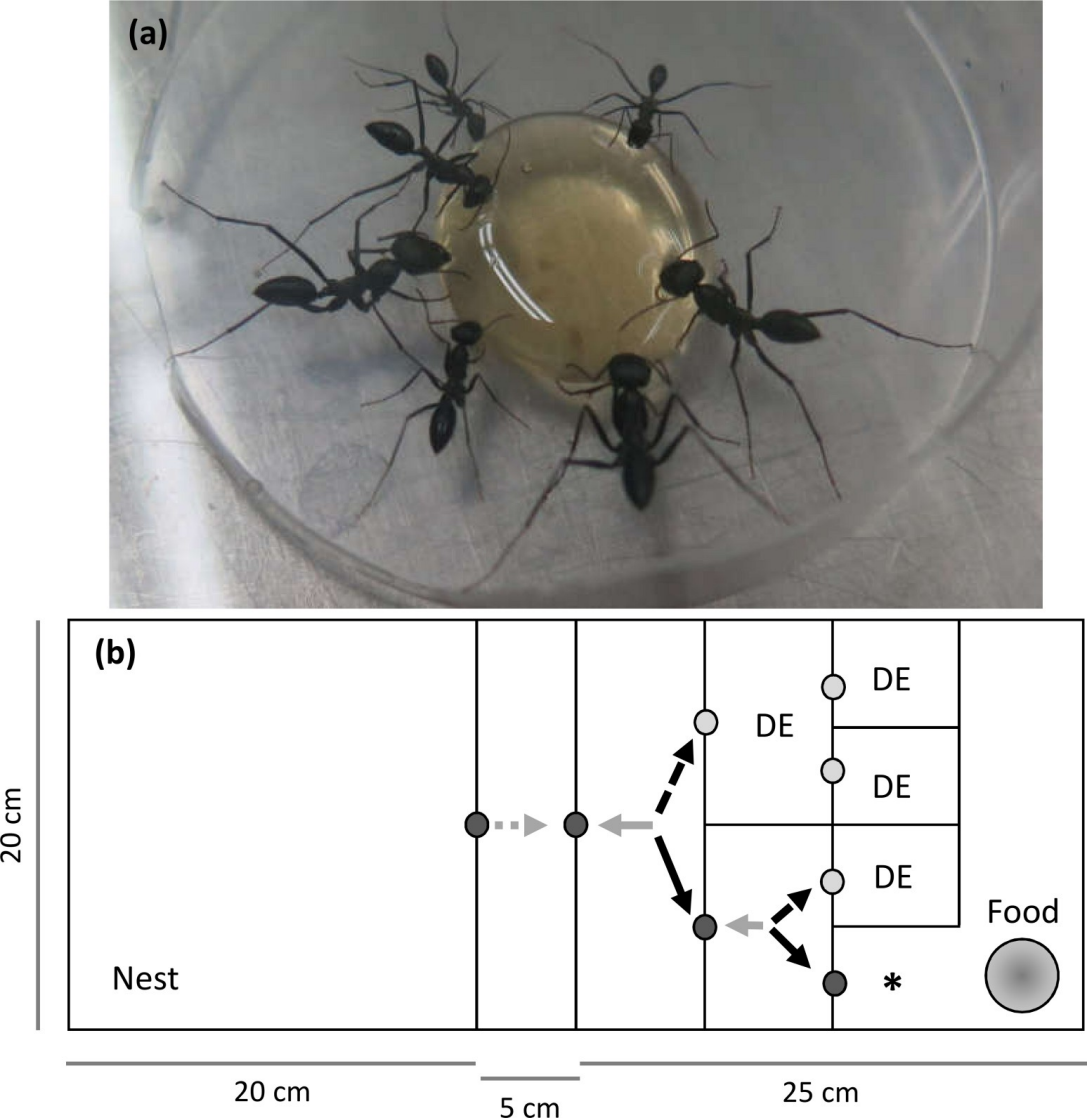
A photo of the studied species and a scheme of the maze used. **(a)** A photo (by MS) of *C*. *niger* workers feeding on the food reward (honey). (**b**) A scheme of the Plexiglas maze used in the experiment. The nest is the complete square on the left and the honey (the food reward) is on the right. In order to reach the food, the workers were required to leave their nest (dashed grey arrow), and enter the narrow corridor leading to the maze. Maze entries equal the number of workers leaving the nest. Workers could move only through passages (small dark or light grey circles). Correct passages (dark grey circles) led to the food reward (black continuous arrow), while wrong passages led to a dead-end (DE, dashed black arrow). Both latter movements are considered forward movement, against the nest direction, while moving back to the nest direction is marked with a continuous grey arrow. Moves back from visited dead-ends are not included in the counting. Maze-solving time was documented as the time required for the first worker to enter the cell containing the food reward (marked with ‘*’).

We let *C*. *niger* colonies search for a food reward in a binary-tree maze (25×20×5 cm; Fig 1). A binary-tree maze comprises repeating sub-units with the same possible moves in each sub-unit. In such mazes, the amount of information required to solve the maze is equal to the number of correct moves from its entrance to the solution [70] (two correct moves, in our case, with a move being a change in the worker’s location between maze cells; Fig 1B). Each nest was connected to a maze through a passage and the ants were allowed to enter the maze and search for food. After each run, all workers in the maze were returned to the nest and the maze was cleaned with ethanol. This procedure was repeated three times, at 30 min intervals, to allow the workers that had found food to come into contact with other workers in the nest (hereafter, the “control set-up”). Three runs with 30 min in-between are sufficient to produce a large improvement in maze-solving time in the same species [65]. Each run continued for 10 min after the first worker had solved the maze and reached the food reward (hereafter, “maze-solving time”; Fig 1B). The food reward comprised 0.5 g honey placed in a 6-cm Petri dish. All runs were videotaped.

We documented six variables (Table 1): (1) “*C*”, the number of moves forward in the correct direction (the opposite direction of the nest), that advanced the workers towards the discovery of the food reward; (2) “*W*”, the number of moves forward but in the wrong direction, leading to a dead-end; (3) “*B*”, the number of moves back to the nest; (4) “maze entries”, the number of workers entering the maze; (5) “*MST*”, maze-solving time, or the time taken for the first worker to solve the maze and reach the food reward; and (6) “workers feeding”, the number of workers feeding on the food reward 10 min following maze discovery. Note that we only measured movements and not “decisions”. We do not possess tools to examine any process in the ant worker’s brain, and each evidence for change in the spatial information or learning is indirect. The process of spatial learning is beyond the scope of this study, and could be either immediate (i.e., when a worker knows the direction to the food reward, it makes no longer any mistakes) or gradual (i.e., even when a worker knows the direction to the food reward some, mistakes are possible).

**Table 1:** A list and definition of the terms used in the manuscript.

Term	Definition
*C* (correct moves)	The number of moves forward in the correct direction, in the opposite direction to the nest, advancing the workers towards the discovery of the food reward.
*W* (wrong moves)	The number of moves forward but in the wrong direction, leading to a dead-end.
*B* (backward moves)	The number of moves back to the nest.
*K* (spatial information)	The correct moves, leading to the food reward, vs. all forward moves, or the sum of correct and wrong moves, leading to a dead-end ($K=\frac{C}{C+W}$)
*M* (forward movement)	The ratio of moves forward in the maze, against the nest direction, and all moves ($M=\frac{C+W}{C+W+B}$)
Maze entries (*ME*)	The number of workers leaving the nest and entering the maze.
Maze-solving time (*MST*)	The time required for the first worker to solve the maze and reach the food reward.
Change in maze-solving time	The difference in maze-solving time between the 3^rd^ and 1^st^ run in the maze (*MST*_*t = 3*_ *–MST*_*t = 1*_)
*L* (spatial learning)	The difference in spatial information between the 3^rd^ and 1^st^ run in the maze (*K*_*t = 3*_ *–K*_*t = 1*_)
Change in maze entries	The difference in maze entries between the 3^rd^ and 1^st^ run in the maze (*ME*_*t = 3*_ *–ME*_*t = 1*_)
Change in forward movement	The difference in forward movement between the 3^rd^ and 1^st^ run in the maze (*M*_*t = 3*_ *–M*_*t = 1*_)

### Definitions of spatial learning and search intensity

We define spatial information (*K*) as the ratio of information known by the workers out of all the information relevant to solving the maze. This can be intuitively defined as $K=\frac{C}{C+W}$, or the ratio between the moves forward in the correct direction and the sum of the latter plus the number of moves forward in the wrong direction. Spatial learning (*L*) is the change in information with successive runs in the same maze: $L=\Delta K=\Delta \left(\frac{C}{C+W}\right)$. In the absence of information, *K* is 0.5, because the probability of moving in the correct direction equals the probability of moving in the wrong direction. When the worker has complete information on the maze, *K* is 1, because the number of moves forward in the wrong direction is zero. The process of spatial learning (*L*), or an increase in *K* with successive runs in the same maze, should shorten the maze-solving time. We assume that the workers know the nest’s direction (based on evidence in other *Cataglyphis* species [71–73]). This does not mean that workers do not go back in the nest’s direction from time to time for different reasons (e.g., failure in locating food), but this is an indication of a lower search intensity. We estimate search intensity in two ways (Table 1): (1) the tendency to move forward in the maze, hereafter “forward movement” (“*M*”), which is the ratio of moves forward in the maze against the nest’s direction and all other moves ($M=\frac{C+W}{C+W+B}$); and (2) the maze entries by workers (the number of workers entering the maze during each run). Higher values of *K*, *M* and maze entries, independently of each another, should lead to faster maze solving.

### Treatments

The three main treatments comprised: (a) the “control set-up” (see “Basic experimental design” above); (b) “a removal treatment”: removing workers immediately after they had reached the food reward and keeping them separately until the end of the experiment; and (c) “a late removal treatment”: removing workers that had reached the food reward but only after marking them, placing them back in the nest, and allowing them to interact with their nestmates for 30 min. Marking was applied to each worker’s gaster, by gently touching it with a marking pen while it was feeding, and without lifting it. We observed no overt responses to marking from the workers. We expected the late removal treatment to discover the food faster with successive runs than the removal treatment, owing to elevated search intensity (triggered by sharing food collected by the workers prior to their removal), but not to spatial learning. In other words, if we observe faster maze solving in the late removal treatment than in the removal treatment, it may suggest that workers transfer non-spatial information on the presence of food nearby. In the control, we expected to detect both elevated search intensity and spatial learning, leading together to faster maze solving. Note that while the same workers could solve the maze in the control during the three runs, in the removal and late removal treatments, each worker could solve the maze only once, and then it was removed. We also applied: (d) “a prior information treatment”. Before the experiment commenced, we first exposed five random workers of each colony to the food reward on a separate plate, outside the nest, without any directional information on the reward location. We then returned them to the nest for 30 min before allowing workers of the same colony to search for food in the maze. This is the only treatment in which the manipulation took place before the experiment commenced, in contrast to the other treatments. This treatment was expected to induce higher search intensity for food and, by doing so, lead to faster discovery of food, without information on its exact location.

Our experiment focuses on the performance of the whole colony rather than that of individual workers, because we did not identify individual workers and the data we obtained refer to the number of events (e.g., workers entering the maze, reaching the food reward, moving forward, etc.). The reason for this is that findings at the colony level are more relevant to colony fitness, while processes taking place at the individual level may or may not remain influential at the colony level (e.g., [74]). Moreover, social insects are often referred to as “super-organisms”, because all units of the colony cooperate to spread their genes [75], and therefore processes at the colony level are no less important than those at the individual level. Finally, there is a growing interest in whole colony behavior and its consistency (reviewed in [76]).

### A small experiment to rule-out the attraction to the food reward by smell

The goal of this small additional experiment was to examine whether workers are attracted to the honey reward by smell. If this holds true, then spatial learning may play a smaller role than we suggest here, and the workers find the honey reward simply because they are attracted to it by smell. We collected additional 14 colonies and let them search for an empty 6-cm Petri plate (no food reward) in the binary-tree maze described above. We define maze-solving time as the time required to find this empty Petri dish. This small experiment was conducted identically to the control set-up, but only with a single run. We compared the results to the first run of the control.

### Statistical analysis

First, we examined whether a correlation exists between maze-solving time and the number of workers feeding using a Pearson correlation test. The link was indeed strong (see Results). We therefore focused on maze-solving time and did not further analyze the other response variable. We then compared the control to the two treatments of the removal and the late removal. Here we were most interested in the change in the response variables across runs, because the manipulation (removal of workers) took place after each run. Next, we compared the first run of the prior information treatment to that of the control. We focused on the first run, because the manipulation (exposure to the food reward) took place before the first run, and we expected its effect on maze-solving time to be the strongest immediately after it had taken place.

Spatial information (*K*) and forward movements (*M*) are both ratios, and some of the values are based on a low sample size (when workers moved only a few movements). We used Wilson's correction for both variables [77], which helps to avoid extreme values, resulting from low sample size:

$$p_{corrected}=\frac{p+\frac{1.96^{2}}{2n}}{1+\frac{1.96^{2}}{n}}$$

*p* is the ratio (*K* or *M* in our case) and *n* is the number of movements in the maze. In order not to use a rule-of-thumb for the minimal sample size for which we applied the correction, we applied it for all ratios.

Maze-solving time was log_10_-transformed and maze entries and the number of workers feeding were square-root-transformed, due to their deviations from a normal distribution. Statistics were conducted in Systat, v. 13 (San Jose, CA), except for the bootstrap (see below), which was conducted in MATLAB (2017).

#### Removal and late removal vs. control

We used four separate repeated-measures ANCOVA to test for the joint effect of treatment and run on the maze-solving time, spatial information, forward movements, and maze entries. We did so in order to examine differences in maze-solving time among treatments and to explain these differences if detected. We took colony size into account as a covariate, because this often affects the number of workers foraging [55, 78–79]. We then calculated the change in maze-solving time (Δ*MST*), spatial learning (*L* = Δ*K*), forward movements (Δ*M*), and maze entries (Δ*ME*) by subtracting the third run from the first run. Negative values for the Δ*MST* indicate that the maze was solved faster with successive runs, while positive values for *L*, Δ*M*, and Δ*ME* indicate improvements with runs. We used the bootstrap procedure (10,000 replications; [80]), separately for each variable and treatment, to calculate the 95% confidence intervals and to examine whether a change had occurred across runs. If the confidence intervals did not overlap with zero, this indicated that the maze was solved faster with successive runs, i.e., that learning had taken place, forward movements had intensified, or more workers had entered the maze.

#### Prior information vs. control

We first used an ANCOVA to test for the effect of treatment on maze-solving time, and then tested separately for the effect of treatment on spatial information, forward movement, and maze entries. We took colony size into account as a covariate.

#### No reward vs. control

We used an ANCOVA to test for the effect of the existence of a reward (control with a 0.5 honey reward vs. an empty plate, no reward) on maze-solving time. Colony size was taken into account as a covariate.

## Results

### The link between maze-solving time and the number of workers feeding

There was a tight negative link between maze-solving time and the number of workers feeding, indicating that the faster the maze is discovered the greater is the number of workers feeding on the food reward 10 min later (1^st^ run: r = -0.310, P = 0.017, n = 59; 2^nd^ run: r = -0.354, P = 0.006; 3^rd^ run: r = -0.555, P < 0.001). Due to this tight correlation, later analysis focused on maze-solving time as the response variable.

### Removal and late removal vs. control

**Fig 2:**
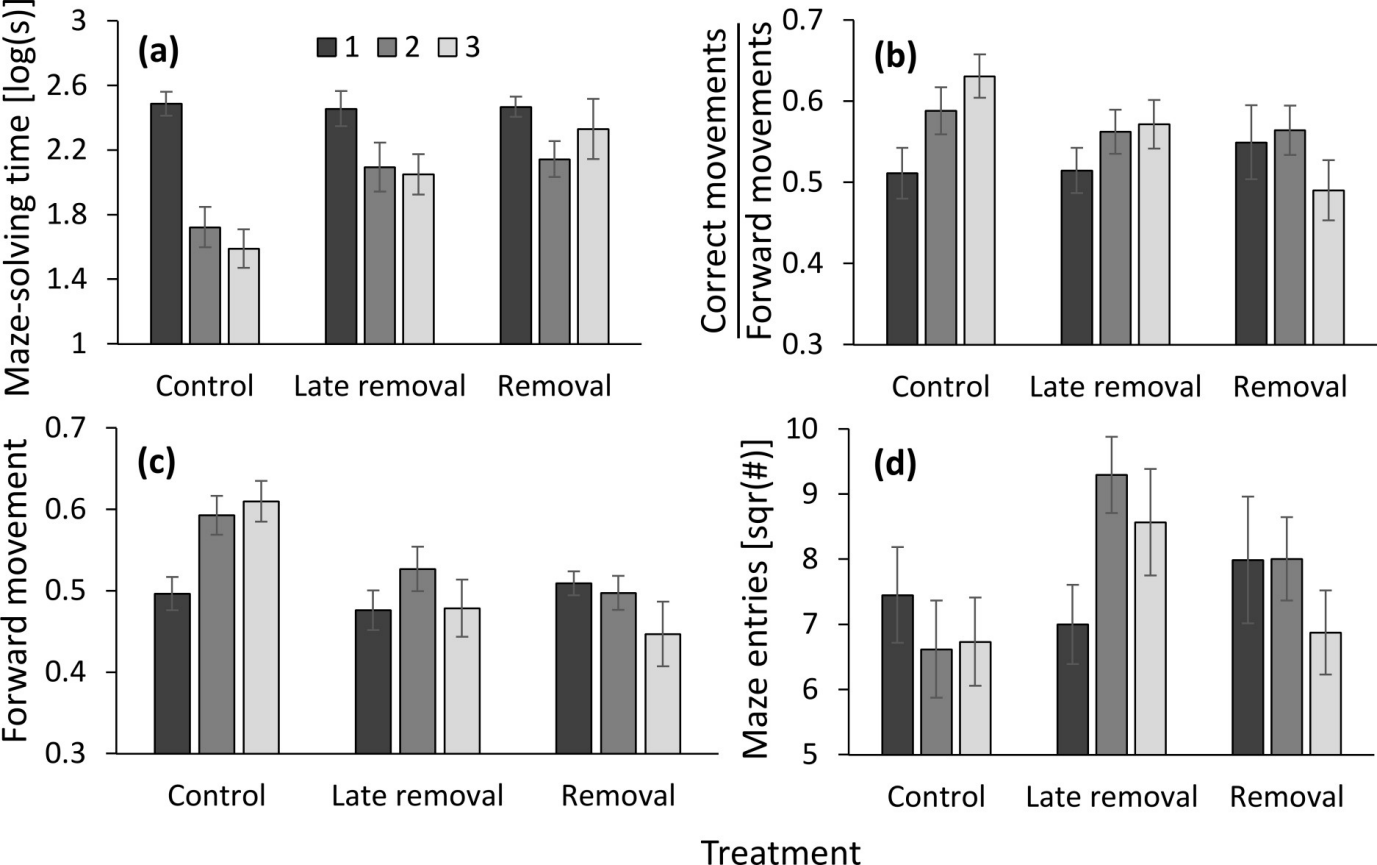
The effect of treatment (removal or late removal vs. control) over three runs (1, 2 and 3) on (**a**) maze-solving time (*MST*); (**b**) the ratio of correct movements to forward movements (defined by us as spatial learning); (**c**) the ratio of moves forward in the maze against the nest’s direction and all other moves (forward movements); and (**d**) maze entries. Means ± 1 SE are presented.

Treatment interacted with the number of run to affect maze-solving time (*MST*): the latter decreased most steeply in the control and the least steeply in the removal treatment. The late removal treatment demonstrated an in-between difference in maze-solving time (Table 2; Fig 2A). Treatment interacted with the number of run to affect the spatial information (*K*), with the largest increase in information with run (or learning) demonstrated in the control, the lowest in the removal treatment, and the late removal was again in-between (Table 2; Fig 2B). Forward movements demonstrated a similar pattern (Table 2; Fig 2C), while there was also a positive effect of colony size on forward movements. Finally, treatment interacted with the number of run to affect the number of maze entries: while entries remained more or less constant or slightly decreased in the control and the removal treatment, they were higher in the second and third run than in the first run in the late removal treatment (Table 2; Fig 2D).

**Table 2:** Results of four repeated-measures ANCOVAs on maze-solving time (*MST*), spatial information (*K*), forward movements (*M*), and maze entries (*ME*), as the response variables. Treatment and colony size are the between-subjects explanatory variables and run number, as well as its interaction with treatment and colony size, are the within-subjects explanatory variables. Treatments include the control, removal and late removal.

	MST	K	M	ME
Treatment	F_2,40_ = 5.709, **P = 0.007**	F_2,40_ = 0.754, P = 0.477	F_2,40_ = 4.253, **P = 0.021**	F_2,40_ = 1.425, P = 0.253
Colony size	F_1,40_ = 2.323, P = 0.135	F_1,40_ = 0.114, P = 0.737	F_1,40_ = 6.155, **P = 0.017**	F_1,40_ = 3.395, P = 0.073
Run	F_2,80_ = 5.175, **P = 0.008**	F_2,80_ = 1.267, P = 0.287	F_2,80_ = 1.774, P = 0.176	F_2,80_ = 0.442, P = 0.644
Run × Treatment	F_4,80_ = 3.199, **P = 0.017**	F_4,80_ = 2.985, **P = 0.024**	F_4,80_ = 6.252, **P < 0.001**	F_4,80_ = 3.458, **P = 0.012**
Run × Colony size	F_2,80_ = 0.469, P = 0.628	F_2,80_ = 0.345, P = 0.709	F_2,80_ = 0.064, P = 0.938	F_2,80_ = 0.183, P = 0.833

The bootstrap procedure indicated that the maze was solved faster with successive runs in the control and late removal treatments, but not in the removal treatment (Table 3). An increase in the spatial information (or spatial learning) with runs took place, as expected, only in the control (Table 3). Forward movements increased and decreased with runs in the control and the removal treatment, respectively. The number of maze entries increased with runs only in the late removal treatment (Table 3).

**Table 3:** Means and 95% confidence intervals for the difference in maze-solving time (*ΔMST*), spatial information (*L* or *ΔK*), forward movements (*ΔM*), and maze entries (*ΔME*) between the third and the first runs. Significant results (when confidence intervals do not include zero) are marked in bold, and indicate a change (either an increase or a decrease) with successive runs.

	Δ*MST*	*L or* Δ*K*	Δ*M*	Δ*ME*
Control	**-0.896** [-1.160, -0.598]	**0.120** [0.053, 0.189]	**0.114** [0.067, 0.163]	-0.717 [-1.847, 0.397]
Late removal	**-0.407** [-0.689, -0.082]	0.057, [-0.026, 0.136]	0.002 [-0.069, 0.070]	**1.570** [0.193, 2.997]
Removal	-0.138 [-0.501, 0.245]	-0.059, [-0.148, 0.020]	**-0.062** [-0.125, -0.003]	-1.109 [-3.300, 1.116]

### Prior information vs. control

**Fig 3:**
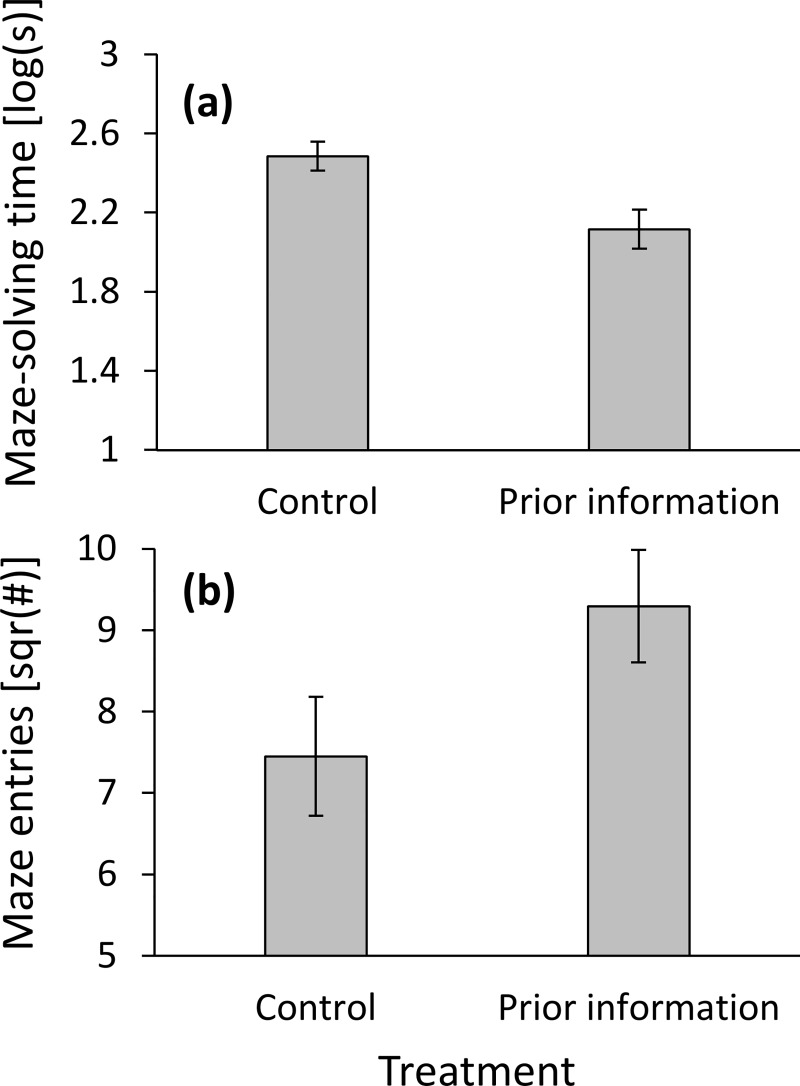
The effect of treatment (prior information vs. control) on (**a**) maze-solving time (*MST*); and (**b**) maze entries (number of workers leaving the nest). Means ± 1 SE are presented.

Maze-solving time was shorter in the prior information treatment than in the control (F_1,28_ = 9.715, P = 0.004; Fig 3A). Colony size had no effect on maze-solving time (F_1,28_ = 3.043, P = 0.092). The reason for the difference in maze-solving time could not be due to a difference in spatial information (*K*) and, as expected, it did not differ between both treatments (F_1,28_ = 2.000, P = 0.168). Forward movement too did not differ between both treatments (F_1,29_ = 0.007, P = 0.932). Colony size affected neither spatial information nor forward movement (F_1,28_ = 0.051, P = 0.824; F_1,28_ = 0.996, P = 0.327; respectively). Colony size had a strong effect on maze entries (F_1,28_ = 9.382, P = 0.005), which were somewhat higher for the prior information treatment than for the control (F_1,28_ = 3.957, P = 0.057; Fig 3B). This is probably why the maze was solved faster in the prior information treatment.

### A small experiment to rule-out the attraction to the food reward by smell

Neither the presence of the honey reward (F_1,27_ = 1.026, P = 0.320) nor colony size affected maze-solving time (F_1,27_ = 0.003, P = 0.958).

## Discussion

When animals repeatedly search for food in the same habitat, experience increases the probability of finding food, while the time required to do so decreases (e.g., [81–83]). Here, we demonstrate the important contribution of search intensity in addition to that of spatial learning to improving food discovery with experience. Specifically, the improvement in maze-solving time following an increase in information on the presence of food nearby was unrelated to spatial learning. The mechanism behind the faster maze-solving may be that of the higher number of workers entering the maze. The fact that faster discovery of food can stem from more intensive searching rather than spatial learning has been noted before, as a result, for example, of a higher hunger level. Our study is nevertheless important in demonstrating that non-spatial information on the presence of food is transferred among individually foraging ant workers and probably elevates search intensity, thereby shortening the time required to discover food.

In the control, when colonies were allowed to repeatedly search for the food reward in the maze, maze solving was faster with successive runs and spatial information increased, demonstrating spatial learning. Faster maze solving is possible also without spatial learning, and we present two pieces of evidence for the contribution of non-spatial information on the presence of food, to maze-solving time. First, exposing workers to the food reward prior to the experiment led to faster maze solving compared to workers, which were not previously exposed to the food reward (prior information treatment vs. the control). This held true, although both the workers in the treatment and those in the control had never encountered the maze before their first search and, therefore, had no spatial information on the maze. As expected, spatial information did not differ between the two groups. The tendency of more workers to enter the maze in the prior information treatment may explain the faster maze solving in this treatment. Second, the comparison between the late removal and the removal treatments indicated that when workers transfer information on the presence of food to other workers, this is sufficient to induce a higher search intensity, even in the late removal treatments, when those workers that had previously found food are not allowed to search again in the maze. We therefore suggest that exposure to food not only elevated the search intensity of those workers that had encountered food, but also that of other workers which were probably fed by them. We suggest that this information transfer affects the workers’ motivational state, as motivation is not only influenced by an animal’s physiological state, but also by the expectation an animal has for the consequences of its activities, such as the further discovery of food [84]. Different aspects of motivation (and not only physiological state) need therefore to be accounted for when studying learning [41, 85]. *Cataglyphis* desert ants are individual foragers, which do not use pheromones to recruit other workers to a discovered food resource [63–65]. This foraging strategy fits small colonies or those searching for food that is randomly distributed in space and is relatively quickly depleted [86–88]. However, a previous study has demonstrated that workers of the studied ant species can transfer information on the presence of food that triggers other workers to leave the nest and commence foraging [89]. This is probably what took place in our experiment too, leading to elevated search intensity and consequently faster maze solving. In other individually foraging social insect species, the return of workers to the nest after successfully finding food stimulates other workers to leave the nest and forage [90–91]. Faster discovery of food is important, especially for individually foraging ant species that cannot dominate large food sources. For example, in two co-occurring harvester ant species competing for the same food, the individually foraging species discovers food faster, while the recruiting ant species dominates the food source and depletes it more efficiently [92]. In general, social insects possess a behavioral flexibility that is clearly superior to that of solitary species. For example, while solitary insects, when starved, can only increase their foraging activity, social insects can respond on two levels: each worker can forage longer, or more workers can leave the colony to forage [93–95].

The treatments providing the colonies with non-spatial information on the presence of food had no effect on the forward movement (*M*), evident in the similar forward movement values in the removal and the late removal treatments, and in the prior information and the control during the first run. Our analysis suggests that even when workers know that food is present nearby, they do not move forward more than they do in the absence of such information. The fact that the workers occasionally returned back in the nest’s direction is supported by a previous study suggesting that “self-avoiding random search” explains better the search mechanism of the same ant species in a maze than “depth-first search” [55]. Self-avoiding random search allows revisiting cells in the maze, while depth-first search does not. It could be that returning to the same area and re-checking it increases the likelihood of detecting food that was missed on the first visit. The only case of increasing forward movement in the present study was in the control with successive runs, which enabled spatial learning (*L*). This suggests that when workers have spatial information where the food is, they move further away from the nest. This supports the explanation that workers occasionally move back when they do not possess any spatial information, in order to search again for food in already visited areas, closer to the nest. Workers from larger colonies moved more forward than those from smaller ones. This is perhaps related to the finding that workers from larger colonies of social insects often forage over larger distances than those from smaller colonies [96–98]. Workers were not attracted to the honey reward by smell, as the *MST* did not differ when honey was offered or not during the first run. This supports our conclusion that spatial learning is an important component in our experiment and that the workers do not reach the honey faster simply because they smell it. It could be that workers learn to associate the honey smell with the food reward. This possibility should be verified in future research by using odorless food reward instead of honey.

Some of the unexplained variance in our experiment can perhaps be related to the changes that take place in the average movement speed with experience [27, 99], which was not measured. Our next step should be to mark and follow individual workers’ behavior in greater detail (e.g., to also document movement speed and tortuosity of individual ants) and to apply different levels of maze complexities. Following the workers that are fed by the workers that had discovered food would conduce to more accurate quantification of the effect of the transfer of non-spatial information among workers, on search intensity. Habitat complexity, in general, and maze complexity, in particular, can affect the time required to reach food [55, 100–101]. It will be intriguing to determine whether elevated search intensity, as presented here, will have the same contribution to maze solving when searching in a more complex maze. Furthermore, examining under which circumstances workers use each of the three mechanisms to solve the maze faster (spatial learning, forward movement, and workers entering the maze) is also of interest. It could be, for example, that in some scenarios spatial learning is harder to achieve and then workers compensate with the two other mechanisms. In conclusion, our take-home message is that search intensity can strongly influence maze solving; and that ant workers, even those considered as individual foragers, transfer information on the presence of food. This information leads to a higher search intensity and faster discovery of food, as a parallel process to spatial learning.

## Supporting information

S1 Dataset (XLSX)
